# Kentucky Asylum for Feeble-minded Children

**Published:** 1860-07

**Authors:** 


THE KENTUCKY ASYLUM FOR FEEBLE-MINDED CHILDREN.—The Kentucky Legislature, emulating the older States in the extent and liberal endowments of its charities, has, by an act at its last session, established an asylum for feeble-minded children, upon the same basis as her lunatic hospitals. This is as it should be. There is no difference whatever in the ultimate objects of asylums for the idiotic and for the insane; requiring the same general principles for their guidance, the same professional skill for the successful management of their inmates, they ought to occupy the same medical position.

A man receives a blow upon the head, causing injury to the brain, and insanity follows; a child falls from its nurse's arms, receiving a like injury, and an arrest of development and idiocy result. If not identical, the closest relationship exists between them. The Institution is under the superintendence of Dr. James Rodman, who is eminently qualified to fill the post which has been intrusted to him.

